# Examining the unidimensionality of the PHQ-9 with first responders: Evidence from different psychometric paradigms

**DOI:** 10.4102/ajopa.v6i0.165

**Published:** 2024-12-10

**Authors:** Tyrone B. Pretorius, Anita Padmanabhanunni

**Affiliations:** 1 Department of Psychology, Faculty of Community and Health Sciences, University of the Western Cape, Cape Town, South Africa

**Keywords:** depression, PHQ-9, confirmatory factor analysis, exploratory factor analysis, parallel analysis, Mokken analysis, unidimensionality, first responders

## Abstract

The Patient Health Questionnaire-9 (PHQ-9) is an effective tool for identifying depressive disorders in diverse populations, making it a valuable resource in both clinical practice and research. However, the factor structure and dimensionality of the instrument have been contested. Studies have raised questions about whether the PHQ-9 adequately captures a single underlying construct or reflects multiple distinct dimensions of depression. This study examines the factor structure of the PHQ-9 among South African first responders using exploratory factor analysis (EFA), confirmatory factor analysis (CFA) with ancillary bifactor indices, parallel analysis and Mokken analysis. A cross-sectional study design was used with data collected from police officers (*n* = 309) and paramedics (*n* =120). Although the EFA identified a two-factor structure, this was not supported by the other analyses. While the one-factor, correlated two-factor and bifactor models of the PHQ-9 had comparable fit indices, the one-factor model appeared to be marginally superior in the CFA. Ancillary bifactor and parallel analysis also did not support the interpretation of the PHQ-9 as multidimensional. Lastly, Mokken scale analysis confirmed that the PHQ-9 is a strong and reliable unidimensional scale of depression. These findings suggest that the PHQ-9 predominantly measures a single construct of depression, consistent with the unidimensional view of the disorder.

**Contribution:**

The present study provides evidence from different measurement perspectives that the commonly used PHQ-9 measures a single construct of depression and not two separate components as some studies suggested. In practice, this simplifies the interpretation of scores, allowing clinicians to assess overall depression severity without needing to differentiate between symptom types.

## Introduction

Depression is one of the most prevalent mental health conditions and a leading cause of disability globally. It is characterised by persistent feelings of sadness, hopelessness and a lack of interest or pleasure in daily activities. In more severe cases, depression can lead to thoughts of self-harm or suicide (American Psychiatric Association, 2022). Depressive disorders have a significant impact on an individual’s ability to function in various aspects of life, including personal relationships and their occupation. Moreover, recent studies suggest that the prevalence of depression is increasing (Moreno-Agostino et al., 2021; Shorey et al., 2022).

In a systematic review and meta-analysis of the literature on adolescent depression, Shorey and colleagues concluded that 34% of adolescents globally were at risk of developing clinical depression (Shorey et al., 2022). Similarly, Hu and colleagues, in their meta-analytic study on depression among older adults, reported a prevalence rate of 28.4%, while Gutiérrez and colleagues, in their systematic review, reported a prevalence rate of 21% among community samples (Gutiérrez-Rojas et al., 2020; Hu et al., 2022). Studies have also highlighted the prevalence of depression among vulnerable population groups including postpartum women (Liu et al., 2022), healthcare workers (Li et al., 2021), teachers (Ozamiz-Etxebarria et al., 2021) and college students (Wang et al., 2023). These findings underscore the substantial and pervasive burden of depressive disorders and highlight the critical need for effective screening practices. Early identification of depressive symptoms through comprehensive screening is essential for timely intervention. This can significantly improve outcomes by preventing the progression of the disorder and mitigating its impact on individuals’ overall well-being.

Although depression has traditionally been approached as a categorical condition – where individuals either meet the criteria for a diagnosis or do not – there is growing evidence to suggest that depression may be better understood as a dimensional phenomenon. This perspective posits that depressive symptoms exist on a continuum, varying in severity from mild to severe, rather than as discrete categories. Growing recognition of the dimensional nature of depression has led to the development of instruments designed to assess both the presence and severity of symptoms (Bianchi et al., 2022).

The Patient Health Questionnaire (PHQ) was specifically developed to facilitate a criteria-based diagnosis of depression, aligning with established clinical guidelines (Kroenke et al., 2001). The instrument comprises nine items that correspond to the diagnostic criteria for major depressive disorder (MDD) as outlined in the Diagnostic and Statistical Manual of Mental Disorders (American Psychiatric Association, 2022). This includes both cognitive-affective symptoms, such as mood disturbances, feelings of guilt or worthlessness and difficulties in concentration, as well as somatic symptoms, including changes in sleep and appetite, fatigue and suicidal ideation (American Psychiatric Association, 2022).

Since its inception, the psychometric properties of the PHQ-9 have been examined across numerous studies, and the instrument has consistently demonstrated strong diagnostic accuracy and reliability (El-Den et al., 2018). The psychometric properties of the PHQ-9 have been assessed in women experiencing perinatal depression, patients with psychiatric disorders, people with multiple sclerosis and patients suffering from major depressive disorder (Beard et al., 2016; Patrick & Connick, 2019; Sun et al., 2020; Wang et al., 2021). These studies have confirmed that the PHQ-9 is an effective tool for identifying depressive disorders in diverse populations and settings, making it a valuable resource in both clinical practice and research. However, research into the factor structure and dimensionality of the instrument has raised questions about whether it adequately captures a single underlying construct or multiple distinct dimensions of depression (Bianchi et al., 2022; Doi et al., 2018).

Using confirmatory factor analysis (CFA), Doi and colleagues examined the factor structure of the PHQ-9 across different groups, including adults diagnosed with MDD, adults with both MDD and a co-occurring anxiety disorder and those without any psychiatric disorder (Doi et al., 2018). The authors reported that the bifactor model was supported in both the clinical and nonclinical samples. Beard and colleagues (2016) conducted a comprehensive validation of the PHQ-9 in a psychiatric sample, using exploratory and confirmatory factor analyses. Their findings suggested a two-factor solution, with one factor capturing cognitive and affective symptoms and the other reflecting somatic symptoms. Using Mokken analysis, Boothroyd and colleagues (2019) concluded that a one-factor solution was viable for the PHQ-9 in a sample of adults, while González-Blanch and colleagues (2018), using CFA, found that both the one-factor and two-factor models provided adequate fit in a primary care setting. A systematic review of studies using CFA to examine the factor structure of the PHQ-9 identified four models including a one-factor solution, a two-factor solution, a bifactor model and a three-factor model (Lamela et al., 2020). Given the ongoing debates about whether the PHQ-9 should be interpreted as measuring a single construct of depression or whether it reflects distinct but related dimensions (e.g., somatic and cognitive-affective symptoms), further research is warranted to clarify its factor structure. The current study aims to contribute to the literature by investigating the dimensionality of the PHQ-9 in a sample of South African first responders using CFA, ancillary bifactor indices, parallel analysis and Mokken analysis.

First responders are routinely exposed to a wide range of stressful and traumatic events during the course of their work. This increases their risk of adverse mental health outcomes. The most common trauma-related disorders encountered among first responders include post-traumatic stress disorder, anxiety and depression. A clearer understanding of the factor structure of the PHQ in this population group may aid in early identification of symptoms of psychological distress and promote targeted interventions.

## Methods

Data were analysed using IBM^®^ Statistical Package for Social Sciences (SPSS) for Windows, version 29, IBM Amos for Windows, version 28 and R, version 4.3.1 (R Development Core Team, 2020) and the package Mokken, version 3.1.2 (Van der Ark, 2012).

### Participants and procedure

Participants consisted of police officers (*n* = 309) and paramedics (*n* = 120) in the Western Cape, South Africa. We constructed an electronic version of the PHQ-9 using Google Forms, and with the approval of administrators of Facebook groups that consisted of first responders, we posted a link to the questionnaire on those sites. In addition, student assistants visited several police stations and hospitals to recruit additional participants.

The majority of the sample were men (45%) and married (51.5%). The mean age of the sample was 39 years (standard deviation [s.d.] = 9.9), and the mean number of years as a first responder was 13.2 years (s.d. = 9.7).

### Instruments

As part of a broader study focusing on the mental health of university students, participants completed a brief demographic questionnaire as well as the PHQ-9. The PHQ-9 consists of nine items to which participants respond using a 4-point scale that ranges from 0 (*not at all*) to 3 (*nearly every day*). An example of an item of the PHQ-9 is ‘Over the last 2 weeks, how often have you been bothered by poor appetite or overeating?’ Higher scores on the PHQ-9 reflect higher levels of depression. The PHQ-9 was validated in two studies, one involving primary care patients and the other obstetrics-gynaecology patients, and Cronbach’s alpha for the two studies was 0.89 and 0.86, respectively. The PHQ-9 has also been used in South Africa with different population groups, for example chronic care patients (Bhana et al., 2015: α = 0.76), an isiXhosa version with adolescents (Rakshasa-Loots et al., 2023: α = 0.87) and tuberculosis patients (Kigozi, 2020: α = 0.84).

### Data analyses

The internal consistency of the PHQ-9 was examined using IBM^®^ SPSS for Windows, version 29 (IBM Corp., Armonk, New York, United States [US]). This included Cronbach’s alpha and McDonald’s omega, the range of interitem correlations, the average interitem correlation, the item-total correlations and the factor loadings for a forced one-factor solution. It is recommended that alpha and omega should ideally be ≥ 0.80 for acceptable reliability (Clark & Watson, 2019). Interitem correlations should be between 0.15 and 0.85 (Paulsen & BrckaLorenz, 2017). A correlation lower than 0.15 reflects that the items come from different content domains, while higher than 0.85 indicates item redundancy. Clark and Watson (2019) recommend that the average interitem correlation should fall within the range 0.15 to 0.50, which would reflect a good homogenous item set. Item-total correlations should be greater than 0.50 (Paulsen & BrckaLorenz, 2017) and factor loadings greater than 0.55 (DeVon et al., 2007), which would indicate that, to a large extent, the items contribute to the measurement of the latent variable.

We also used SPSS to conduct an exploratory factor analysis (EFA; principal components analysis with varimax rotation). To examine the extent to which the data are suitable for factor analysis, the Kaiser–Meyer–Olkin (KMO) measure of sampling adequacy and Bartlett’s test of sphericity were conducted. Items would be considered sufficiently correlated to conduct factor analysis if Bartlett’s test was significant and KMO was greater than 0.50.

Confirmatory factor analysis was conducted using IBM^®^ Amos for Windows Version 28 (IBM Corp., Armonk, New York, US). In the CFA, we examined three models of the factor structure of the PHQ-9: a one-factor model, a correlated two-factor model and a bifactor model. A one-factor model assumes that the nine items of the PHQ-9 load on a single unidimensional scale, whereas a correlated two-factor model assumes that two factors that are correlated are an adequate representation of the PHQ-9. A bifactor model assumes that a general factor (total scale) and two uncorrelated specific factors (subscales) are an adequate representation of the PHQ-9. The fit indices that were used to assess model fit were χ^2^, which ideally should be nonsignificant, although a nonsignificant χ^2^ would be indicative of a perfect fit (Jöreskog et al., 2016), the goodness-of-fit statistic (GFI), the Tucker–Lewis index (TLI) and the comparative fit index (CFI). For the last three indices, a value of ≥ 0.95 is indicative of an acceptable fit (Hu & Bentler, 1999). We also included the root mean square error of approximation (RMSEA) and a value ≤ 0.08 is considered an acceptable fit (MacCallum et al., 1996), while an RMSEA value of ≤ 0.05 is indicative of good model fit (Byrne, 2013). In addition to these commonly used indices, we included the Akaike information criterion (AIC), which is a model comparison index, and models with lower AIC values are considered better-fitting models. In general, factor loadings in CFA greater than 0.50 are regarded as acceptable (Saptono, 2017).

Fit indices provide an indication of whether a particular structure fits the data, but they do not address the dimensionality of a particular scale: that is, whether any specific factors that were examined in the CFA explain a sufficient amount of variance beyond that explained by the general factor. Ancillary bifactor indices enable the examination of the amount of variance explained by both the general and specific factors (Rodriguez et al., 2016). The minimum bifactor indices needed to draw conclusions about the dimensionality of an instrument are explained common variance (ECV), omega hierarchical (ω*H*) and percentage of uncontaminated correlations (PUC). These indices were obtained using a freely available online Excel calculator (Dueber, 2017). Explained common variance is the proportion or percentage of item variance explained by the general and specific factors. An ECV > 0.70 for the general factor would indicate that the instrument in question is essentially unidimensional (Rodriguez et al., 2016), as this would indicate that the general factor accounts for 70% of item variance. OmegaH is an estimate of the variance in raw total scores that is accounted for by the general and specific factors. In the case of specific factors, ω*H* reflects the proportion of variance in the raw total scores after separating out the variance explained by the general factor. In general, ω*H* of the specific factors (ω*H*_*s*_) is used to determine whether the specific factor has added value beyond the general factor, and, in this regard, it is suggested that specific factors should not be interpreted if ω*H*_*s*_ < 0.50 (Gignac & Watkins, 2013). Percentage of uncontaminated correlations reflects the proportion of covariances between items that is accounted for by the general factor, and a PUC > 0.80 reflects a very strong latent variable (Rodriguez et al., 2016). An additional index included was the construct replicability coefficient *H*, which provides an indication of the reliability of a latent factor and how well the observed variables (items) represent the underlying factor. An *H* value > 0.80 indicates a latent variable that is well defined (Dueber, 2017).

We conducted parallel analysis, using SPSS syntax that is freely available online (O’Connor, 2000), to confirm the minimum number of factors that represent the factor structure of the PHQ-9. In the parallel analysis, a large number of datasets (1000) with the same number of variables and observations are simulated, and the actual eigenvalues obtained in the current study are compared to the 95th percentile of the simulated eigenvalues. Only eigenvalues of the actual dataset that are greater than the 95th percentile of the eigenvalues from the simulated datasets represent meaningful factors.

Lastly, we conducted a Mokken scale analysis (MSA), which is a nonparametric item response theory psychometric method, using the package ‘Mokken’ (Van der Ark, 2012) in R software (R Development Core Team, 2020). We used the monotone homogeneity model in MSA, which assumes unidimensionality and monotonicity. Mokken scale analysis uses an automated item-selection procedure (AISP) to determine whether items are unscalable (indicated by a zero) as well as the number of scales that the items load on (as many values as there are scales). If all the items have an AISP value of 1, all of the items load on a single scale, thus demonstrating unidimensionality. Mokken scale analysis also provides an index of the strength of the scale, referred to as the *H*-coefficient, and the strength of each item’s contribution to the measurement of the latent variable, referred to as a *H*_*i*_-coefficient. With respect to the *H*-coefficient, Wind (2017) provides the following rule of thumb for evaluating the strength of a scale: *H* greater than 0.50 = strong scale, *H* between 0.40 and 0.50 = moderate scale and *H* less than 0.40 = weak scale. With regard to the individual items, an *H*_*i*_ less than 0.30 reflects items that do not fit well and do not significantly contribute to the measurement of the latent variable (Mokken, 2011).

Monotonicity in MSA refers to the assumption that the probability of endorsing an item is nondecreasing over increasing values of the latent variable (Wind, 2017). Mokken scale analysis provides an index, called a *Crit* value, to determine whether the assumption of monotonicity has been violated. *Crit* values greater than 80 are considered serious violations, while values less than 80 are considered minor and acceptable. In addition, violations can also be assessed using the #vi function, which identifies violations, and the #zsig function, which is the significance of a *z*-test indicating which of the identified violations are significant. Mokken scale analysis also provides a reliability coefficient for the scale, *MS*_*rho*_.

## Results

The reliability of PHQ-9 can be considered satisfactory, as both Cronbach’s alpha and McDonald’s omega were 0.89. The interitem correlations, descriptive statistics, item-total correlations (ITC) and factor loadings are presented in Table 1.

**TABLE 1:** Internal consistency indices for the Patient Health Questionnaire-9.

PHQ-9 items	1	2	3	4	5	6	7	8	9
1. Anhedonia	-	-	-	-	-	-	-	-	-
2. Depressed mood	0.62**	-	-	-	-	-	-	-	-
3. Sleep disturbance	0.51**	0.57**	-	-	-	-	-	-	-
4. Fatigue	0.49**	0.44**	0.64**	-	-	-	-	-	-
5. Appetite changes	0.46**	0.49**	0.59**	0.57**	-	-	-	-	-
6. Low self-esteem	0.44**	0.53**	0.41**	0.32**	0.47**	-	-	-	-
7. Concentration difficulties	0.52**	0.46**	0.44**	0.40**	0.45**	0.61**	-	-	-
8. Psychomotor disturbances	0.41**	0.51**	0.39**	0.29**	0.44**	0.55**	0.60**	-	-
9. Suicide ideation	0.37**	0.50**	0.37**	0.23**	0.42**	0.60**	0.51**	0.60**	-
Mean	1.19	1.10	1.22	1.34	1.16	0.99	1.03	0.82	0.65
s.d.	0.94	1.01	1.01	0.98	1.03	0.97	1.00	0.93	0.90
ITC	0.65**	0.70**	0.67**	0.57**	0.66**	0.66**	0.68**	0.64**	0.61**
λ	0.73**	0.78**	0.75**	0.66**	0.74**	0.75**	0.76**	0.73**	0.70**

ITC, item-total correlation; PHQ-9, Patient Health Questionnaire-9; s.d., standard deviation.

** , *p* < 0.001.

Table 1 indicates that the interitem correlations (0.23 to 0.64) and the average interitem correlation (0.48) were within the recommended range, thus indicating that the items reflect the same content domain and there were no redundant items. The ITC correlations ranged between 0.57 and 0.70 and were all above 0.50. Similarly, the factor loadings ranged between 0.66 and 0.78 and were all above 0.55. The ITC and factor loadings confirm that all items contribute to the measurement of the latent variable. In general, all the indices in Table 1 support the internal consistency of the PHQ-9.

Kaiser–Meyer–Olkin was greater than 0.50 (0.89), and Bartlett’s test was significant (*p* < 0.001), thus confirming the suitability of the data for factor analysis. The results of the EFA are reported in Table 2.

**TABLE 2:** Results of exploratory factor analysis.

PHQ-9 items	Factor 1 Cognitive-affective	Factor 2 Somatic
1. Anhedonia	0.41	**0.64**
2. Depressed mood	0.53	**0.58**
3. Sleep disturbance	0.25	**0.82**
4. Fatigue	0.07	**0.87**
5. Appetite changes	0.35	**0.70**
6. Low self-esteem	**0.78**	0.27
7. Concentration difficulties	**0.71**	0.35
8. Psychomotor disturbances	**0.80**	0.22
9. Suicide ideation	**0.83**	0.15

Note: Values in bold indicate primary factor loading.

PHQ-9, Patient Health Questionnaire-9.

Table 2 shows that the EFA resulted in two factors, similar to previous factor analysis, and they were labelled similarly. However, it is also noticeable that four items cross-loaded with loadings above 0.32 (Tabachnick et al., 2013). With the exception of the factor loading of the item ‘fatigue’ on the cognitive-affective factor, all the factor loadings and cross-loadings were statistically significant.

**FIGURE 1:**
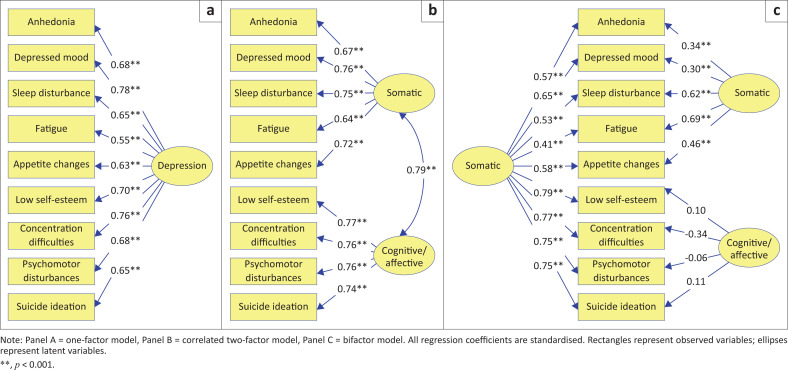
Three models of the factor structure of the Patient Health Questionnaire-9.

The three models of the PHQ-9 that were examined with CFA, namely the one-factor, the correlated two-factor and the bifactor models, are presented in Figure 1. The CFA fit indices are reported in Table 3.

**TABLE 3:** Fit indices for three models of the factor structure of the Patient Health Questionnaire-9.

Fit index	Good fit criteria	One-factor	Correlated two-factor	Bifactor
*χ* ^2^	-	53.35	69.16	68.93
*df*	-	16	20	18
*p*-value	Nonsignificant	< 0.001	< 0.001	< 0.001
GFI	≥ 0.95	0.97	0.97	0.97
TLI	≥ 0.95	0.96	0.95	0.95
CFI	≥ 0.95	0.98	0.97	0.97
RMSEA	≤ 0.08	0.07	0.08	0.08
90% CI	-	0.05, 0.01	0.06, 0.10	0.06, 0.10
AIC	Lower values	111.35	119.16	122.93

*df*, degree of freedom; GFI, goodness-of-fit; TLI, Tucker–Lewis index; CFI, comparative fit index; RMSEA, root mean square error of approximation; CI, confidence interval; AIC, Akaike information criterion.

Table 3 indicates that all three models fit the data to an acceptable degree (GFI, TLI, CFI ≥ 0.95, RMSEA ≤ 0.08), and AIC indicated that the one-factor model was marginally the best model. Figure 1 shows that the factor loadings for the one-factor model all exceeded 0.50 and ranged between 0.55 and 0.78. Similarly, the factor loadings for the correlated two-factor model ranged between 0.64 and 0.77. However, the two factors were strongly associated (0.79), suggesting that a two-factor structure is redundant. Although the fit indices for the bifactor model showed an acceptable fit, the loadings for the two subscales were problematic. For the somatic subscale, three of the loadings were below 0.50, and for the cognitive-affective subscale, all of the loadings were nonsignificant and two were negative.

The results of the ancillary bifactor analysis overwhelmingly indicated that the PHQ-9 is essentially unidimensional:

- In terms of ECV, the general factor accounted for 73.3% of the variance of all items, while the specific factors explained 26.7% (somatic factor = 24.1%, cognitive-affective factor = 2.6%).
- ω*H*_*s*_ of the two specific factors was below 0.50 (0.241 and 0.026).
- The construct replicability coefficient (*H*) of the general factor was greater than 0.80.
- When PUC, ECV and ω*H* of the general factor are considered together, PUC was lower than 0.80 (0.56), ECV was greater than 0.60 (0.73) and ω*H* was greater than 0.70, which would indicate that there is some multidimensionality (the ECV of the somatic factor was 0.24), but this was not strong enough to override the conclusion that the PHQ-9 is essentially unidimensional.

The unidimensionality of the PHQ-9 was also confirmed by parallel analysis. In this regard, a principal component analysis (PCA) of the current dataset identified only one eigenvalue (4.83) that was greater than the 95th percentile (1.29) of a range of simulated eigenvalues. The second eigenvalue in the current dataset (1.16) was lower than the 95th percentile (1.20) of the simulated eigenvalues.

The results of the Mokken analysis are reported in Table 4.

**TABLE 4:** Results of the Mokken analysis.

PHQ-9 item	AISP indicator	*H*_*i*_	s.e.	Monotonicity		
				*Crit*	#vi	#zsig
1. Anhedonia	1	0.52	0.03	0	0	0
2. Depressed mood	1	0.55	0.03	0	0	0
3. Sleep disturbance	1	0.53	0.03	0	0	0
4. Fatigue	1	0.47	0.03	0	0	0
5. Appetite changes	1	0.52	0.03	0	0	0
6. Low self-esteem	1	0.53	0.03	0	0	0
7. Concentration difficulties	1	0.54	0.03	0	0	0
8. Psychomotor disturbances	1	0.53	0.03	0	0	0
9. Suicide ideation	1	0.52	0.03	0	0	0

PHQ-9, Patient Health Questionnaire-9; AISP, automated item selection procedure; s.e., standard error of *H*_*i*_; #vio, violations of monotonicity; #zsig, significance of violations of monotonicity.

Table 4 indicates that all of the PHQ-9 items loaded on a single scale, as indicated by the AISP value of 1. The *H*-coefficient of the unidimensional PHQ-9 and *MS*_*rho*_ were 0.52 and 0.90, respectively, reflecting a very strong and reliable scale. The *H*_*i*_ values of the individual items were all above 0.30 and ranged between 0.47 and 0.55. There were no violations of monotonicity, as confirmed by the *Crit* value as well as #vio and #zsig.

## Discussion

There is ongoing debate regarding the dimensionality of the PHQ-9, with studies reporting various factor structures, ranging from unidimensional models to more complex multifactor solutions (Lamela et al., 2020). Some studies have identified a two-factor model, typically distinguishing between cognitive-affective and somatic symptoms, while others have proposed three- or four-factor models (Beard et al., 2016; Bianchi et al., 2022). The variability in findings highlights the need for further research of the underlying structure of the PHQ-9 from a variety of perspectives. Against this backdrop, the current study used exploratory and confirmatory factor analyses as well as ancillary bifactor indices, parallel analysis and Mokken analysis to examine the dimensionality of the PHQ-9 in a sample of South African first responders. There were several important findings.

Firstly, the PHQ-9 demonstrated satisfactory internal consistency, further underscoring its reliability as a measure of depressive symptoms. The high interitem correlations and ITC values confirmed that the scale items cohesively represent the same underlying construct without redundancy. This is particularly relevant in both theoretical and practical contexts, as it affirms that the PHQ-9 accurately captures a comprehensive range of depressive symptoms. The reliability of the instrument confirms that it can be used to screen for depression, track symptom changes over time and inform treatment decisions.

Secondly, the results of the EFA align with previous studies, identifying two distinct factors within the PHQ-9, which were labelled similarly to earlier research (Beard et al., 2016; Doi et al., 2018). However, the presence of cross-loadings suggests that certain items may tap into multiple dimensions of depression simultaneously, complicating the interpretation of the two-factor model. The identification of these cross-loadings is important, as it raises questions about the clear delineation of cognitive-affective and somatic symptoms within the PHQ-9, potentially indicating an overlap that needs to be considered in both research and clinical applications.

Thirdly, the CFA further explored the dimensionality of the PHQ-9 by comparing three models: a one-factor model, the correlated two-factor model and the bifactor model. The results indicated that while all the models provided an acceptable fit, the one-factor model was marginally superior. This finding supports the notion that depression, as measured by the PHQ-9, may be best understood as a unidimensional construct, where all items contribute to a single underlying factor. The correlated two-factor model, despite having acceptable fit indices, revealed a high correlation between the two factors, similar to the high correlation reported by Boothroyd and colleagues (2019). This strong association suggests that distinguishing between cognitive-affective and somatic symptoms may be redundant, as both factors appear to be closely intertwined. This finding challenges the utility of a two-factor model, reinforcing the idea that depression symptoms may not neatly divide into separate categories but rather exist along a continuum that is best captured by a single dimension. The bifactor model, although showed an acceptable overall fit, presented significant issues with the loadings on the subscales. The problematic loadings, particularly the nonsignificant and negative loadings on the cognitive-affective subscale, indicated that the bifactor model does not provide a coherent or meaningful representation of the data, further contesting the case for more complex factor structures.

Further support for the unidimensionality of the PHQ-9 came from the results of the ancillary bifactor indices and parallel analysis. The fit indices of the bifactor model confirmed a dominant general factor explaining 70% of the item variance, while the two specific factors did not add value beyond the variance extracted by the general factor. Similarly, the parallel analysis found only one eigenvalue that was greater than the 95th percentile of a range of eigenvalues that was simulated over 1000 datasets, thus confirming that one factor is sufficient to account for the factor structure of the PHQ-9.

Finally, the results of the Mokken analysis further confirmed the unidimensionality of the PHQ-9, with all items loading on a single strong and reliable scale. This reinforces the interpretation of the PHQ-9 as a measure of a single, cohesive construct of depression, rather than a collection of disparate symptoms.

In terms of theoretical implications, these findings suggest that the PHQ-9 predominantly measures a single construct of depression, consistent with the unidimensional view of the disorder. This supports the theoretical perspective that depressive symptoms, whether cognitive-affective or somatic, are manifestations of the same underlying condition, rather than distinct dimensions. In practice, the confirmation of unidimensionality has important implications for the use of the PHQ-9 in clinical and research settings. It simplifies the interpretation of scores, allowing clinicians to assess overall depression severity without needing to differentiate between symptom types. From a theoretical standpoint, these findings support the notion that depression can be reliably measured through a set of diverse yet interrelated symptoms, as operationalised in the PHQ-9. This consistency aligns with established theories of depression, which conceptualise it as a multifaceted disorder encompassing cognitive, affective and somatic components. The solid factor loadings across all items further strengthen this perspective, indicating that each item is a meaningful reflection of the latent depression construct.

The study had certain limitations. The cross-sectional design of the study limits the ability to draw causal inferences. Future studies using longitudinal designs could provide more comprehensive insights into the stability and predictive validity of the PHQ-9. Furthermore, the reliance on self-reported data may introduce response biases, such as social desirability or recall bias, which could affect the accuracy of the findings. Although the PHQ-9 is widely used and validated, the incorporation of clinician-administered assessments or corroborative data from other sources could enhance the robustness of future studies. The sample was limited to first responders, which may affect the generalisability of the findings to other populations. First responders are exposed to unique stressors and traumatic events that may influence the expression of depressive symptoms differently compared to the general population or other occupational groups. Participants also came from one province in South Africa, which also limits generalisability.

## Conclusion

This study confirms the reliability and validity of the PHQ-9 as a unidimensional measure of depressive symptoms among first responders in South Africa. The strong psychometric properties observed, including internal consistency, factor structure and item functioning, support its use as an effective tool for assessing depression in this high-risk occupational group.
